# Air pollution and alveolar health

**DOI:** 10.1183/16000617.0280-2024

**Published:** 2025-09-03

**Authors:** Carmelo Sofia, James G.H. Parkin, Joseph A. Bell, Lareb S.N. Dean, Liam J. Edgeway, Lucy Sayer, Natasha H.C. Easton, Donna E. Davies, Ben G. Marshall, Stephen T. Holgate, Luca Richeldi, Mark G. Jones, Matthew Loxham

**Affiliations:** 1 School of Clinical and Experimental Sciences, Faculty of Medicine, University of Southampton, Southampton, UK; 2 Faculty of Medicine and Surgery, Università Cattolica del Sacro Cuore, Rome, Italy; 3 NIHR Southampton Biomedical Research Centre, University Hospital Southampton, Southampton, UK; 4 Institute for Life Sciences, University of Southampton, Southampton, UK; 5 Fondazione Policlinico Universitario A. Gemelli IRCCS, Università Cattolica del Sacro Cuore, Rome, Italy; 6 Both authors contributed equally

## Abstract

Exposure to air pollution has been associated with up to 9 million premature deaths per year worldwide, with the respiratory system a key site for its effects. Air pollution exposure is a well-established risk factor for the development and exacerbation of airways diseases and lung cancer, however relatively little is known regarding the risks associated with air pollution interacting with areas of gas exchange – the alveoli and pulmonary interstitium. In recent years, evidence has emerged identifying a role in the development and progression of sub-clinical interstitial lung abnormalities as well as progression and risk of exacerbation of fibrotic interstitial lung diseases. This review outlines the epidemiologic evidence that air pollution perturbs alveolar health. It considers the different components of ambient air pollution, how penetration to the alveoli is determined by particle size and whether the response to alveolar exposure may be modulated by personal susceptibility factors. We discuss potential acute and chronic pathogenic mechanisms of injury upon the pulmonary interstitium and how these may contribute to the development and/or progression of interstitial processes. Finally, we explore current knowledge gaps and the potential for air pollution interventions in vulnerable individuals to support alveolar homeostasis and so prevent disease development and/or progression.

## Shareable abstract

**Emerging evidence suggests air pollution may be associated with abnormalities and disease of the alveoli and pulmonary interstitium. This review examines this evidence alongside mechanisms of alveolar injury, susceptibility factors and knowledge gaps.** https://bit.ly/3GsTO1E

## Introduction

Air pollution exposure is a known risk factor for adverse health outcomes, including all-cause and respiratory mortality. Although the association is strongest for the respiratory and cardiovascular systems, there is a rapidly accumulating body of evidence that acute and chronic exposure to air pollution is potentially associated with effects on almost every organ in the body and, consequently, with a large number of diseases [1, 2]. The World Health Organization (WHO) estimates that the combined effects of ambient air pollution and household air pollution lead to approximately 6.7 million premature deaths annually, particularly in low- and middle-income countries [3]. However, other recent studies have suggested that this number may be higher, approaching 9 million [4, 5]. Notably, the Global Burden of Disease Study has found that exposure to airborne particulate matter (PM) represents the single leading risk factor for ill health [6]. Studies examining the economic burden of air pollution identify a significant cost from medical care. The World Bank calculated a cost of USD 8.1 trillion, or over 6% of total global gross product, in 2019 [7]. In England, approximately GBP 5.56 billion is expected to be spent between 2017 and 2025 on the National Health Service and social care for supporting healthcare linked to air pollution [8].

Inhalation is the main route of exposure to air pollutants; thus, the respiratory system represents one of the primary targets for health issues [9, 10]. There is an established association for air pollution exposure with triggering of acute exacerbations of airway diseases, especially for upper airways [11, 12], with more recent evidence for an association with incident asthma [13, 14]. For instance, PM with aerodynamic diameter <2.5 μm (PM_2.5_) (also known as fine PM) and ground-level ozone (O_3_) are associated with increased rates of asthma exacerbation [15], increased children's hospital admissions and emergency department visits related to exacerbations [16, 17], and increased asthma mortality [18]. Short- and long-term air pollution exposure is a leading risk factor for increased incidence and mortality in COPD, even after adjustment for major mortality risk factors such as cigarette smoking [4, 19, 20]. Moreover, indoor and outdoor ambient air pollution exposure, together with cigarette smoking and radon exposure [21], represent a risk factor for lung cancer [22, 23], the leading cause of cancer-related deaths worldwide [24].

Remarkably, little is known regarding the risks associated with air pollution penetrating to areas of gas exchange – the alveoli and pulmonary interstitium. Interstitial lung diseases (ILDs) comprise a heterogeneous group of diseases characterised by the proliferation and thickening of the pulmonary interstitium, whilst the term interstitial lung abnormality (ILA) refers to specific computed tomography findings in patients without clinical suspicion of ILD and can represent a precursor to development of clinically relevant ILD [25, 26]. In recent years, a number of studies have proposed an association between ILAs and ILDs and air pollution exposure including disease induction and progression. This association may vary depending on pollutant–gene interactions (*i.e.* the interaction of exposure to pollutant(s) and underlying hereditary risk factors depending on genotype) as well as on other susceptibility factors and the mechanisms are not well understood. However, there is a need to better understand the effects of air pollution on the pulmonary interstitium, in order to mitigate impacts on disease development and progression.

This review outlines the epidemiologic evidence that air pollution perturbs alveolar health. It considers the different components of ambient air pollution, how penetration to and across alveoli is determined by physicochemical properties of particles or gases, and whether the response to alveolar deposition may be modulated by personal susceptibility factors. We discuss potential acute and chronic pathogenic mechanisms of injury upon the pulmonary interstitium and how these may contribute to the development and/or progression of interstitial abnormalities and disease. Finally, we explore current knowledge gaps and the potential for air pollution interventions in vulnerable individuals to support alveolar homeostasis and so prevent disease development and/or progression.

## Ambient air pollution composition and air data monitoring

Ambient air pollution consists of PM, organic species including polycyclic aromatic hydrocarbons and volatile organic compounds, as well as specific gaseous molecules including carbon monoxide (CO), ozone (O_3_), nitrogen dioxide (NO_2_) and sulfur dioxide (SO_2_), which are associated with adverse health effects [27, 28]. Air pollution sources can be both anthropogenic, meaning human-derived (for example industry, traffic and combustion), and natural (wildfires, erosion, *etc*.), which are dramatically increasing due to anthropogenic climate change. Pollutants can be also described as primary (released at source) or secondary (formed from ageing and reactions between other pollutants).

PM refers to airborne solid and liquid particles, comprising a complex mixture of components such as elemental carbon (EC; soot), organic carbon, metals and secondary components formed from reactions between primary emissions (*e.g.* sulfates, nitrates, ammonium and chloride). Sources of primary PM include geogenic sources such as sand and salt, generated by erosion-like processes and wind movement, sea spray aerosol, and volcanic emissions. Primary PM may also contain carbonaceous particles released following fuel combustion (*e.g.* from diesel exhaust and burning of coal, wood and biomass). There is also increased interest in emissions of ammonia from agriculture (fertiliser and livestock waste) as a precursor to secondary PM [29, 30]. PM is conventionally described according to size, expressed as aerodynamic diameter, up to 10 µm, 2.5 µm or 0.1 µm (PM_10_, PM_2.5_ and PM_0.1_, respectively), or also as coarse (PM_10–2.5_), fine (PM_2.5_) and ultrafine (PM_0.1_) particles, respectively. Recent modelling indicates that, in the UK, approximately 25% of the population-weighted mean PM_2.5_ concentration is primary anthropogenic PM_2.5_ and approximately 20% is secondary inorganic aerosol, with the remainder predominantly of nonanthropogenic origin (which also includes secondary organic sources) [31]. Exposure to PM_10_ and PM_2.5_ has been linked to health effects, although there tends to be more consistent evidence for a greater effect of PM_2.5_ [32].

A related metric of air pollution is black carbon (BC), which is produced when carbonaceous fuel is combusted incompletely, that is when not all the carbon is oxidised to CO_2_ (or CO), and carbon in its elemental form is released. There have been differing definitions as to the precise nature of BC and related measures, but an extensive review by Lack *et al.* [33] suggest that it strongly absorbs visible light in a wavelength-independent manner, vaporises at around 4000 K, contains graphitic sp^2^-bonded carbon and is insoluble in water and common organic solvents. A range of methods are available for BC quantification [34]. Two commonly used methods are the aethalometer, where BC is quantified by measuring transmitted light through particles collected on a filter or tape, allowing online readouts [33, 35] and quantification of EC (which correlates to varying degrees with BC) through thermo-optical or thermo-gravimetric methods [36]. There is a growing evidence base for the health effects of BC, as summarised in a recent US Environmental Protection Agency report [37], although an observed effect may be due to the combustion-associated components such as metals and semi-volatile organic carbon compounds carried by the carbon core [38]. However, the WHO have determined the current evidence base to be insufficient for formulation of guideline values, instead emphasising the need for national and regional monitoring, production of emission inventories, and mitigation steps [27].

Other common ambient air pollutants include gaseous pollutants such as O_3_, NO_2_, SO_2_ and CO. Ground-level (tropospheric) O_3_ is a major component of photochemical smog and is formed through the reaction with other gases in the presence of sunlight. NO_2_ is a reddish-brown gas usually generated from the high temperatures of fuel combustion in processes of transportation, industry and heating. Sulfur dioxide usually derives from the burning of sulfur-rich fossil fuels (coal and oil) and the smelting of mineral ores containing sulfur. CO is a toxic gas produced by the incomplete combustion of carbonaceous fuels, especially from motor vehicles [39, 40].

Additionally, exposure may also occur in the form of occupational pollutants, referring to harmful substances in the workplace. Such exposures may be important determinants of health effects, potentially inducing various forms of pulmonary fibrosis, such as those caused by asbestos fibres and crystalline silica (silicon dioxide) [41].

Effective air pollution monitoring networks have a crucial role in measuring and reporting the concentrations of a range of pollutants, as well as their fluctuations, in turn yielding potential insight into sources. Typically, air quality data can be captured using local air quality monitoring stations, most commonly for NO_2_, often with PM_10_ and PM_2.5_, reflecting interest in monitoring road traffic-associated emissions. These are generally used to monitor compliance with legal limits on air pollution concentrations and generally report readings on an hourly basis. The measurement of air pollutant concentrations at fixed-location monitoring sites is a typical approach used for epidemiologic studies to assess the relationship between exposure to air pollutants and health. However, an inadequate number of monitors outside of major cities and in rural areas in many countries, on account of the cost and maintenance requirements of reference-grade equipment, can result in exposure misclassification of people living a significant distance from a monitoring station [42]. Poor spatial coverage of monitoring networks can be improved upon through use of data from satellite monitoring, albeit with relatively poor temporal resolution, and at a spatial resolution of approximately 1–50 km [43]. Other approaches to quantifying exposure include personal exposure monitoring, mobile monitoring, dispersion models and exposure assessment modelling [44–46]. The accuracy and spatial resolution of estimates may be improved through generation of hybrid models, which integrate data gathered and generated using different approaches at different scales, such as satellite and ground-based monitoring, chemical transport and dispersion modelling, and land-use regression, using data integration platforms to combine different data sources [47, 48].

PM_2.5_ can represent an accurate and robust predictor of mortality in studies of long-term exposure to ambient air pollutants [49, 50]. However, with the exception of personal monitoring, such approaches are generally unable to account for the fact that people spend their time in multiple different environments with spatiotemporally differing pollution characteristics, adding a further source of exposure misclassification. Since there are a great number of variables and confounders, the use of different methods of air quality data collection, as well as the potential for exposure misclassification, may significantly influence epidemiological studies on air pollution associations with health effects, partially explaining existing apparent discrepancies in terms of associations with clinical outcomes. Pollution exposure data based on home address may be inaccurate if environmental exposures away from home (*e.g.* time at school or work) are not considered in the analysis. In addition, the time frame of evaluation of the air pollution exposure needs to be appropriate to provide an accurate estimate of the relationship between acute air pollution exposure and acute outcomes, but also of the risk to the development of a specific disease over time, given that the risk may be associated with cumulative exposure over an extended period.

## Ultrafine PM

**FIGURE 1:**
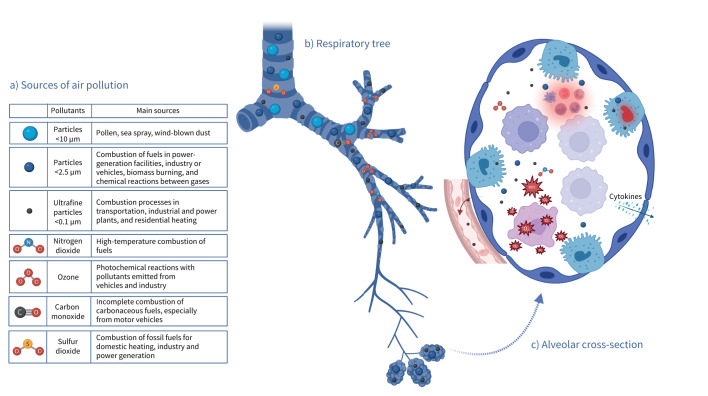
Sources of air pollution at different locations within the respiratory tree. a) Summary of potential sources of air pollution including particulate matter by size fraction, ground-level ozone (O_3_) and nitrogen dioxide (NO_2_). b) Penetration of different air pollution sources identified in a) to sites within the respiratory tree. Both composition and size influence particulate matter penetration, with only particulate matter with aerodynamic diameter <2.5 μm typically capable of reaching the alveolar region. c) Overview of potential mechanisms of alveolar homeostasis perturbation by air pollution sources including oxidative stress, DNA damage, release of proinflammatory mediators, recruitment of inflammatory cells and translocation to the pulmonary capillaries. Figure created in BioRender (https://BioRender.com/mo0n0l0).

Ultrafine particles (UFPs) can penetrate and deposit in the peripheral lung, and there is also evidence for their translocation into the systemic circulation [51, 52]. However, understanding of the effects of ultrafine PM remains poor, partly hampered by lesser understanding of ultrafine PM exposure. There is evidence that ultrafine PM may pose a specific risk on account of its ability to penetrate towards the gas exchange tissues of the lung, increased surface area:volume ratio resulting in greater interaction with extra- or intracellular milieu, increased ability to carry adsorbed toxicants, as well as compositional differences, with smaller particles tending to derive more from combustion than their larger counterparts [53–55]. Ultrafine PM is also able to translocate across the gas–blood barrier formed by the alveolar and capillary membranes, and in doing so enter the circulation (figure 1). Properties which appear important in governing this process are particle size and surface area, and coating of the particle (the corona) from opsonisation and/or surface chemistry following generation [56–60]. It is also important to consider that findings from the world of nanotoxicology, which tends to focus on engineered (*i.e.* intentionally generated) nanoparticles, may be crucial in informing this aspect of ambient ultrafine PM toxicology [61], although the degree of homogeneity of engineered nanoparticles is unlikely to be represented in ambient UFPs.

Compared to PM_10_ and PM_2.5_, there is a relative paucity of research into ultrafine PM. Ultrafine PM is subject to much less monitoring, with no legal or guideline recommendations for their ambient concentrations which, unlike PM_10_ and PM_2.5_, are generally measured as number of particles (n·cm^−3^) rather than as a mass concentration (μg·m^−3^), with number and total surface area potentially being the most health-relevant metrics [62]. Indeed, it is noteworthy that while ultrafine PM contributes a relatively small proportion to the overall mass concentration of airborne PM, it contributes the great majority of the particle number concentration [52, 55, 63]. Significant monitoring challenges arise due to the characteristics of UFPs, including rapid dispersion [64], short atmospheric lifetime [65] and high spatial and temporal variability. UFP concentrations can fluctuate over short distances and timescales, and their sources (such as traffic emissions and other combustion processes) contribute to steep concentration gradients. Furthermore, UFP concentrations exhibit variable degrees of correlation (but generally not strong) with fine PM [66, 67], meaning that simply assuming that PM_2.5_ exposure can be used as a proxy is incorrect. The lack of standardised monitoring methods and regulatory frameworks has hindered the establishment and use of UFP monitoring networks [68], thus large-scale assessments of UFP exposure and associated health risks remain inadequate. In addition, the high spatiotemporal variability of UFP hinders modelling, while the lack of regulated monitoring networks hinders model validation [69]. Nonetheless, there are now moves towards the development and validation of such models, which have the potential to yield valuable new insight into the health effects of exposure to these relatively unknown, but ubiquitous, UFP [69–74].

## Defining the “exposome”

Traditionally, epidemiological studies of associations between exposures and respiratory disease have focused on one single exposure or small number of exposures to define the most relevant risk factors for respiratory diseases. However, a comprehensive characterisation of the environmental contributors is essential to understand the global impact of exposures and reduce the number of false-positive or false-negative findings. This has begun to focus the research on the effect of multiple concurrent exposures on the pathogenesis of a specific disease. The concept of the “exposome’ was introduced by Wild [75] in 2005, defined as the “life-course environmental exposures (including lifestyle factors), from the prenatal period onwards” to highlight the need to characterise a personal environmental exposure throughout life. This approach was at first applied in cancer research. There are three different but complementary domains of exposome research, namely internal (internal biological processes), general external (*e.g.* economic factors and the urban environment) and specific external (local environment) [75]. This particular method can be extended in the field of ILDs, assessing groups of potential exposures using validated biomarkers at different time points throughout the lifetime [10], to detect the most harmful exposures and adopt appropriate preventive measures in susceptible individuals.

## Potential mechanisms of alveolar lung injury

The various types of air pollution travel through the airways of the respiratory tree, reaching the alveoli if they do not either interact with the antioxidant rich airway lining fluid (in the case of gases) [76, 77] or deposit in the upper or lower airways (in the case of particles) (figure 1) [78, 79]. Particle deposition occurs by three mechanisms. Impaction (for particles >5 µm aerodynamic diameter) predominates in the upper airways. Conversely, gravitational sedimentation (predominantly for particles 1–8 µm in diameter) and Brownian diffusion (increasing as particle size decreases and dominating for particles of aerodynamic diameter <0.5 µm) are the dominant mechanisms in the alveoli [80, 81]. Mechanisms by which PM may affect lung health include oxidative stress processes, local and systemic inflammation, DNA damage, telomere shortening, and through epigenomic changes [82–85]. However, mechanisms by which ambient air pollution penetrating to the alveolus contributes to the development and progression of parenchymal lung diseases are poorly understood. Because of the strength of evidence for the burden of ambient PM on health and because of the larger body of evidence on the effects on PM rather than pollutant gases from experimental studies, the following section focuses on PM [86].

### Alveolar epithelium and pulmonary interstitium injuries

**FIGURE 2:**
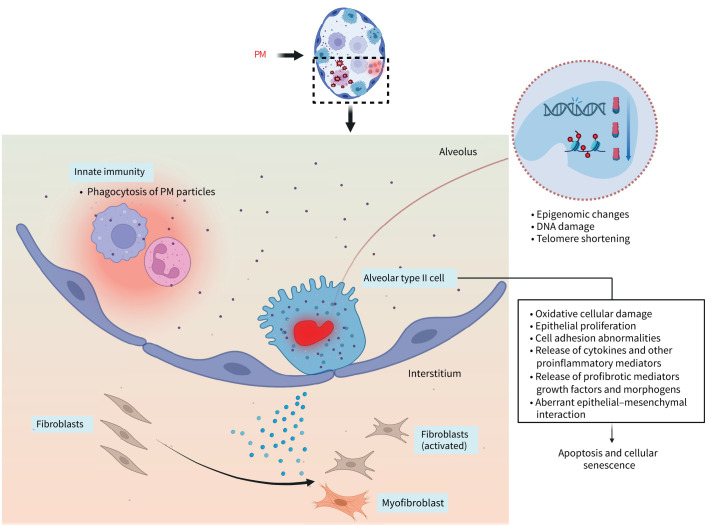
Proposed mechanisms of injury of the alveolus by particulate matter (PM). Innate immune cells are recruited to the alveolus to phagocytose PM particles, whilst oxidative stress, epigenomic alterations and local inflammation may promote type II alveolar epithelial cells to undergo apoptosis and/or cellular senescence. Cumulative exposures may lead to the shortening of telomeres, whilst dysregulation of epithelial–mesenchymal crosstalk may promote activation of fibroblasts and pro-fibrogenic mediators. Figure created in BioRender (https://BioRender.com/pix26ud).

Particles that reach the alveolar region have the potential to influence the microenvironment through a number of mechanisms (figure 2). Evidence suggests that following PM deposition in alveoli, there is upregulation of proinflammatory mediators (cytokines and chemokines) that recruit phagocytic cells, including neutrophils and macrophages, which are able to phagocytose particles [87]. This pro-inflammatory response may be enhanced by the presence of certain metallic and organic components within PM, particle-borne endotoxin and increased particle surface area (as discussed above) [88]. Components including metals can cause oxidative cellular damage through the generation of oxygen-free radicals and reactive oxygen species (ROS). Generating mechanisms may directly involve particle components or occur *via* particle-mediated upregulation of cellular ROS-generating systems such as dual oxidase and NAD(P)H oxidases [89]. Alternatively, mitochondrial dysfunction resulting from particle exposure may result in perturbation of the electron transport chain and increased mitochondrial ROS generation [90, 91]. This, in turn, can induce inflammation and cytotoxic effects when they exceed the antioxidant defence capacity of the lung [92]. PM contributes to cellular mutagenicity through mechanisms including oxidative DNA damage, adduct formation and DNA strand breakage [92, 93]. Smaller particles, particularly within the PM_0.1_ fraction, may infiltrate the lung barrier and increase cellular permeability, inducing a pro-inflammatory condition involving release of damage-associated molecular patterns, such as high mobility group box 1 protein [94]. Interestingly, volunteers with idiopathic pulmonary fibrosis (IPF) and COPD have been found to exhibit increased lung clearance of inhaled ^111^indium-labelled ultrafine carbon particles compared to healthy volunteers, while the translocation of these particles into the blood appears to be greater in volunteers with IPF compared to those with COPD, perhaps indicative of impaired alveolar barrier function facilitating translocation [95, 96].

There is overlap between pathways proposed to underlie the development and progression of pulmonary fibrosis, and those perturbed by acute and chronic ambient air pollution exposure, so supporting the concept that ambient air pollutants may initiate or contribute to the cycle of alveolar injury, disordered repair and fibrosis observed in lung fibrogenesis. Constant or recurrent alveolar type II epithelial cell (ATII) microinjuries by pollution exposure may sustain an aberrant epithelial–mesenchymal interaction in susceptible individuals [97, 98]. Upon injury, ATII cells release profibrotic factors, growth factors and morphogens (*e.g.* sonic hedgehog), and recruit inflammatory cells, resulting in fibroblast proliferation and activation into myofibroblasts. UFPs may be retained within the surfactant layer and can enter alveoli [99], causing stronger and more persistent inflammation than larger particles, with higher alveolar type II cell proliferation, macrophage changes and risk of early interstitial fibrotic foci development [52]. The development of fibrotic alterations is driven by the interaction of injured alveolar epithelial cells and several biochemical mediators [100], such as profibrotic transforming growth factor β (TGF-β), connective tissue growth factor, interleukin (IL)-13, fibroblast growth factor 2, insulin-like growth factor 1, platelet-derived growth factor and others with an antifibrogenic role such as interferon-γ or IL-1 [100–103].

In a study investigating gene expression changes in human embryonic lung fibroblasts exposed to extractable organic matter from ambient air pollution particles [104], TGF-β signalling dysregulation was observed in a dose-dependent manner. This included an upregulation of bone morphogenic protein type-2, an antagonist of TGF-β, and a downregulation of SMAD3, an effector of TGF-β signalling, and the transcription factors DNA-binding inhibitors 1 and 2 [104]. The typical “fibroblastic foci” are the source of excessive and abnormal collagen deposition that then leads to distortion of lung architecture [98, 105]. In early animal studies, chronic and sub-chronic exposure to high ozone levels was associated with elevations in collagen synthesis rates in the lungs [106, 107]. Similarly, recent studies have found increased collagen deposition surrounding the small airways in mice exposed to PM_2.5_, accompanied by increased bronchoalveolar lavage IL-1β and TGF-β1 [108], as well as increased expression of fibronectin and vimentin, markers of epithelial–mesenchymal transition [109].

### Telomere shortening as a marker of ageing

Telomeres are nucleoprotein structures located in all mammals at the end of each chromosome arm. They are formed of highly conserved hexameric (TTAGGG) tandem repeat DNA sequences and are responsible for maintaining genome integrity and functionality [110]. In human cells, telomeres have specific tools to maintain the telomere length (TL) with each cell division, such as the reverse transcriptase telomerase enzyme. However, the system may experience damage whereupon the cell progressively loses its capacity to proliferate, reaching a “replicative capacity” [111]. Evidence suggests critically short telomeres are indicative of cell senescence and that reintroduction of a functional telomerase can restore the cell from senescence [112]. Telomeres normally shorten with increasing age, but the process can be accelerated by chronic inflammation and oxidative stress [113–115].

A study of subjects with high PM exposure in China found a significant association between blood TL and duration of air pollution exposures (personal exposure to PM_2.5_, EC and ambient PM_10_ concentrations from local monitoring stations) [116]. Short-term exposures (same day as examination for personal PM_2.5_ and EC, 1–2 day lag for ambient PM_10_) were associated with increased blood TL, whereas longer exposures (albeit only 14 days and therefore not long-term exposures) were associated with decreased TL. Although no causality was demonstrated, the authors hypothesised that this may reflect a role for transiently increased TL in acute inflammation, followed by shortening of TL due to exhaustion of the cellular response to prolonged pro-oxidant exposures, suggesting a pro-inflammatory and oxidative impact of PM [116]. Another previous study had shown that telomere attrition is associated with chronic exposure to black carbon, an indicator of traffic-related PM_2.5_ [117]. Surprisingly, these results were not recently confirmed in a UK cohort, where a small inverse association between exposure and leukocyte TL disappeared after correction for factors such as smoking and socioeconomic status [118], although this cohort was characterised by a lower level of pollution exposure compared to the Chinese cohort. Similarly, Shull *et al*. [119] did not demonstrate any significant association between TL and PM_2.5_ exposure in 280 fibrotic ILD cases (primarily IPF), with a correlation coefficient of 0.08. Thus, further research is required to establish if leukocyte TL may be indicative of cellular exposure to oxidative stress and inflammation from environmental exposures.

These findings are notable as senescence of alveolar epithelial cells and fibroblasts is emerging as a promoter of pulmonary fibrosis, although the mechanisms are not fully understood. Studies have demonstrated abnormally shortened telomeres in some fibrotic ILDs [120, 121]. The involvement of telomeres in pulmonary fibrosis is also supported by evidence that variations in the genes telomere reverse transcriptase (*TERT*) and telomerase RNA component (*TERC*) are associated with development or prognosis of pulmonary fibrosis [122–124]. Whilst familial associations are identified, there is no genetic explanation for shortened telomeres in ILDs in a number of cases. This suggests the potential for environmental factors to be a significant determinant, with air pollution postulated as a possible aetiology which merits prospective investigation.

### Epigenetic changes

Air pollution exposure is able to effect epigenetic changes, while there is also evidence that epigenetic changes may be associated with ILDs, although direct evidence for epigenetic mechanisms mediating an effect of air pollution exposure on ILDs is lacking [125]. Epigenetic control generally modulates gene expression without modifying nucleotide base sequences and evidence has already shown specific epigenetic alterations in IPF [126]. These include modified DNA methylation (DNAm) patterns, histone modifications, microRNA (miRNA) expression, histone lactylation and noncoding RNA expression. DNAm can occur near genes involved in IPF pathogenesis, thereby affecting their expression [127]. In 2022, Goobie *et al*. [128] evaluated the association between PM_2.5_ exposure (over 3 months and 1 year before blood collection) and global DNAm percentage in peripheral blood DNA samples from patients with IPF, showing an association between increased DNAm and PM_2.5_ exposure for the 3-month exposure period and a similar trend approaching significance for the 1-year exposure period. Interestingly, the study also analysed PM_2.5_ composition and found DNAm associations for PM_2.5_ sulfate and ammonium, suggesting an important role for anthropogenic PM_2.5_. Histone modifications have also been associated with altered apoptotic pathways, observed in animal models of lung fibrosis and in IPF-derived fibroblasts [129]. Moreover, the inhibition of histone deacetylase appears to promote fibroblast apoptosis and prolong survival in bleomycin-injured mice [125, 130]. *In vitro*, it was found that PM_2.5_ exposure in human bronchial epithelial cells can sustain epigenomic changes, such as reduced DNAm and site-specific histone modifications, and the effect is dependent on concentration and exposure duration, especially in cells derived from patients affected by COPD [131]. *In vivo*, two studies in subjects with asthma have demonstrated DNAm modifications at regulatory cytosine–guanine dinucleotide sites of the forkhead box transcription factor 3 locus, implicated in asthma and IPF pathogenesis [132, 133], due to ambient exposures to NO_2_, CO, polycyclic aromatic hydrocarbons and PM_2.5_ [134, 135]. Furthermore, a case–control study in Beijing has examined the relationship between ambient traffic-derived pollutants and circulating histone H3 modifications. In a study population composed of office workers and truck drivers, there was a negative association between short-term PM_10_ exposure and blood concentrations of H3 lysine 27 tri-methylation and H3 lysine 36 tri-methylation, suggesting a role of global histone H3 modifications induced by traffic-related PM exposures [136].

Changes in miRNA expression patterns can also affect gene expression, influencing key cellular functions such as cell growth and differentiation. MiRNAs are noncoding RNAs typically consisting of 17–25 nucleotides [137, 138]. Among all miRNAs, miRNA-21 has been identified as playing a significant role in IPF pathogenesis [139]; others that may be involved include miRNA-182-5p, miRNA 199a-5p and miRNA-31 [140]. Few studies have investigated the effects of air pollution on miRNA expression profiles [141]. Some circulating miRNAs have been found to be associated in a concentration-dependent manner with different pollutant exposures, such as PM_10_, PM_2.5_ and NO_2_, as early as 2 h of exposure [142]. Diesel exhaust particles, a major component of diesel exhaust, can increase miRNA-21 expression in human bronchial epithelial cells [143]. Moreover, seven miRNAs were found to have significantly higher levels in association with PM_2.5_ personal exposure [144]. Another epigenetic change which may be important is histone lactylation, which can directly stimulate gene transcription [145]. Lactate released from myofibroblasts, which show metabolic reprogramming towards a glycolytic metabolic phenotype, as well as in bronchoalveolar lavage fluid from mice with bleomycin-induced lung fibrosis, has been found to modify macrophage histones, promote profibrotic gene expression and induce endoplasmic reticulum stress and apoptosis in A549 cells [146, 147]. Interestingly, PM_2.5_ and crystalline silica have been observed to induce similar glycolytic reprogramming and histone lactylation both *in vivo* and *in vitro*, providing evidence of a possible mechanistic similarity between the two [148, 149].

## Lung parenchymal health effects

In recent years, a number of research studies have investigated the relationship between lung fibrosis and air pollution. Additionally, a potential role of air pollution in the development and progression of ILAs has emerged, raising the intriguing possibility that air pollution may be a factor that determines the progression from sub-clinical ILA to lung fibrosis in susceptible individuals.

### Interstitial lung abnormalities

ILAs are defined as the incidental identification of radiological findings potentially compatible with ILD affecting more than 5% of any lung zone, without previous clinical suspicion of ILD [25, 150]. Differentiation between ILAs and clinical and subclinical ILD is based on clinical evaluation. Risk factors for ILAs include increasing age, tobacco smoke, other inhalational exposures (such as vapours, gases, dusts and traffic-related air pollution) and genetic predisposition [151–153]. In a cohort of 6813 participants enrolled in the Multi-Ethnic Study of Atherosclerosis (MESA) study and followed for 10 years, Sack *et al.* [154] found that exposure to ambient NO_x_ was associated with a higher prevalence of ILAs in nonsmokers. NO_x_ and ambient PM_2.5_ exposure were associated with progression of high-attenuation areas, a recognised radiological marker of subclinical lung fibrosis [155, 156]. Moreover, in the Framingham Heart Study, higher long-term exposure to EC, a constituent of traffic-related PM_2.5_, was associated with ILAs and ILA progression [157].

### Lung fibrosis as a spectrum

IPF, a prototypic progressive fibrotic lung disease, is the most studied parenchymal lung disease in relation to air pollution, with recent evidence suggesting a potential role in development, progression and acute exacerbations of IPF. Very little is known regarding the role of air pollution in other fibrotic lung diseases.

#### Risk of development and progression

The effect of long-term exposure to air pollution (PM_10_, NO_2_ and O_3_) on the risk of IPF incidence was initially evaluated in an Italian study conducted in Northern Italy (Lombardy) between 2005 and 2010, showing that IPF incidence significantly increased in the presence of higher levels of cold season NO_2_, while the association with incidence rate for the overall and the warm season approached significance (p<0.10) [158]. No associations were found with other pollutants such as PM_10_ and O_3_, and the impact of PM_2.5_ was not assessed in this study, which was also unable to correct for smoking status.

More recently, the association between individual air pollutant exposures and incident IPF was investigated in a large-scale prospective cohort study based on data from the UK Biobank [159]. Long-term exposure to NO_2_, NO_x_ and PM_2.5_ was significantly associated with an increased risk of incident IPF. PM_2.5_ showed the highest population-attributable risk, followed by NO_x_ and NO_2_. This risk was not significantly increased in the case of PM_10_ exposure. The authors subsequently found, in the same cohort, that decreased exposure to PM_2.5_ and NO_2_ were potential mediators of an observed inverse association between IPF incidence and residential proximity to greenness (as a measure of green space), albeit with large confidence intervals [160].

In 2019, an observational study by Singh *et al*. [161] showed a positive correlation between hypersensitivity pneumonitis cases and environmental annual mean PM_2.5_ levels, suggesting a possible pollution-associated incidence effect in non-IPF fibrotic ILDs. Moreover, a significant positive association between PM_2.5_ exposure (approximated by annual concentrations) and rheumatoid arthritis-associated interstitial lung disease incidence was found [162]. Examination of specific PM_2.5_ components found a positive association for ammonium, as also found for DNA methylation above (section 5.3) and BC, both suggestive of anthropogenic PM, but also for mineral dust. Interestingly, there was a marked change in PM_2.5_ component-disease associations, especially for mineral dust, switching from negative to positive association, depending on whether single-pollutant Cox proportional hazards models or g-computation was used in the final analysis, as a result of strong (negative in the case of mineral dust) inter-component correlations.

Other studies have investigated the effect of pollution exposure on clinical outcomes. A single-centre study by Winterbottom *et al.* [163] looked at 135 patients with IPF in Pennsylvania and New Jersey and found a significant association between long-term exposure to PM_10_ and disease progression, as measured by decline in forced vital capacity (FVC). Interestingly, although associations with PM_2.5_ were examined in this study, the authors found no associations with IPF, speculating that detection of any association may have been hindered by the greater spatial homogeneity of PM_2.5_ compared to PM_10_ over the area covered by the study population. Nonetheless, within the same population, there was a PM_2.5_-associated increase in use of supplemental oxygen, which was not observed for PM_10_, leading the authors to suggest a potential overall effect of cumulative exposure to ambient PM, rather than any PM size-specific effect. Another paper published in quick succession, by Johannson *et al.* [164] examined associations between weekly FVC and weekly residential-level air pollutant concentrations (O_3_, NO_2_, PM_2.5_ and PM_10_) over a 40-week period in 25 patients in the San Francisco area. Here, unlike the study of Winterbottom *et al.* [163], the authors found no association between air pollution exposure and changes in lung function. However, of note, they did find mean FVC % predicted was associated with concentrations of NO_2_, PM_2.5_ and PM_10_, averaged over the 40-week study period, suggestive of an effect on disease severity but not disease progression. A possible reason for different findings in the latter study, as suggested by the authors, is the small sample size along with the short study period, but nonetheless both studies broadly imply that increased concentrations of air pollution may be associated with deleterious effects in IPF. In line with this, a 2020 systematic review by Harari *et al*. [165] analysed the existing literature concerning the impact of ambient air pollution on IPF and ILD, including seven prospective studies published within the last 6 years (one in 2014, two in 2017, three in 2018 and one in 2019). This concluded there was evidence for a potential link between air pollution exposure and the development and progression of IPF in terms of lung function, acute exacerbation rate, mortality rate and disease incidence. In another, recent, retrospective study of a cohort of 69 patients with IPF, air pollution exposure levels from 2011 to 2020 showed negative effects associations for O_3_, CO, NO_2_ and NO_x_ exposure with clinical, functional and radiological outcomes, regardless of age, sex, smoking status and other factors [166]. Another retrospective study, of patients with systemic sclerosis-associated ILD, average O_3_ exposure in the 5 years prior to diagnosis was associated with the presence of Goh algorithm-defined extensive ILD at diagnosis and with progression at 24 months [167, 168]. In terms of hospitalisations, Liang *et al*. [169] analysed the acute effects of ambient air pollution on hospitalisation risk in patients with IPF in Beijing and found that daily 24 h PM_2.5_ concentrations showed a significant association with IPF hospitalisation rate.

The mean PM_2.5_ concentration over the period of the study of Liang *et al*. [169] was 76.7 μg·m^−3^; however, importantly there is emerging evidence for effects of air pollution exposure on disease progression at relatively low pollution concentrations. A recent study of 570 patients with IPF in Australia found that living within 100 m of a major road was associated with a more rapid decline in oxygen uptake into the alveolar capillaries, measured as diffusing capacity of the lung for carbon monoxide, with identification of an association for PM_2.5_ but not NO_2_. PM_2.5_ 25th, 50th and 75th percentiles were 5.7, 6.8 and 7.9 μg·m^−3^, all of which are below air quality standard annual average concentrations in Australia (8 μg·m^−3^), the European Union (20 μg·m^−3^), USA (9 μg·m^−3^), UK (20 μg·m^−3^) and Canada (8.8 μg·m^−3^), amongst others [170]. Furthermore, the mean modelled pollutant exposure concentrations in the aforementioned UK Biobank studies were 26.65 μg·m^−3^ (NO_2_), 9.99 μg·m^−3^ (PM_2.5_) and 16.23 μg·m^−3^ (PM_10_, only stated in the former study) [159, 160], representing values well within UK annual limit values in force at the time and still applicable at the time of writing, of 40, 20 and 40 μg·m^−3^, respectively. Furthermore, the UK Biobank values are lower than the more stringent contemporaneous WHO guideline values of 40, 10 and 20 μg·m^−3^, respectively. Indeed, it is only since the most recent iteration of these guidelines, in 2021, that the above Biobank study mean concentrations would breach WHO guidelines, which now stand at 10, 5 and 15 μg·m^−3^, respectively. This is in support of the growing evidence base supporting the assertion that even relatively low concentrations of air pollutants are associated with measurable effects on health. The Effects of Low-Level Air Pollution: A Study in Europe (ELAPSE) study in particular is providing growing evidence of such associations, not just with mortality, but also respiratory effects including incidence of asthma and COPD [13, 171, 172]. This, in turn, provides further evidence towards the growing understanding that legally enforceable air pollution limits at both acute and chronic scale do not represent “safe” concentrations. Indeed, there is no such established “safe” concentration of commonly measured/regulated air pollutants, below which associations with health effects are not observed [173–176].

A study across three cohorts found overall increased mortality and transplant rates and worsened baseline FVC associated with increased exposure to PM_2.5_, but inter-cohort differences suggested a potential inflection point of the exposure–response relationship at 8–10 μg·m^−3^ PM_2.5_, as well as a potential importance of PM_2.5_ from industrial and transportation sources [177]. In the first study of its kind, a follow-up by the same group on two of these cohorts found an association between exposure to ultrafine PM (PM_0.1_) and transplantation-free survival, baseline lung function and lung function decline, in a manner sensitive to the accuracy of exposure linkages [178]. This sensitivity to exposure classification method is due to the greater spatial heterogeneity of ultrafine PM compared to PM_2.5_ and emphasises the requirement for development of new exposure models, along with establishment of new monitoring networks (as discussed above) towards the understanding of what may be an important exposure in terms of alveolar health and fibrotic lung disease [179].

#### Risk of acute exacerbation

The association between air pollution exposure and acute exacerbation of IPF has been investigated across different cohorts. A longitudinal study of a Korean cohort of 436 patients with IPF revealed an association between higher O_3_ and NO_2_ exposure in the previous 6 weeks and the development of acute exacerbation [180]. Unfortunately, exposure estimates in this study were only based on regional air quality monitoring data and PM_2.5_ was not evaluated. Another study, evaluating data from air quality monitoring stations closest to patients’ geocoded residences, confirmed that short-term exposure to higher levels of O_3_ can increase the risk of acute exacerbation of IPF [181]. This study also found a significant association between overall mortality and PM_10_ and PM_2.5_ exposure levels [181]. Similarly, a case–control study of 352 patients with IPF within a nationwide registry in Japan, found that there was higher monthly mean exposure to NO and PM_2.5_ in the period preceding acute exacerbation of IPF compared to periods without acute exacerbation, with a lag of 1–2 months between mean exposure and associated exacerbation [182]. An association of acute exacerbations with a longer period of exposure was found in a cohort of 118 patients with IPF in Greece, where the risk of acute exacerbation was associated with mean exposure concentrations for NO_2_, PM_2.5_ and PM_10_ over the year preceding the exacerbation [183].

To date, the aetiology of acute exacerbation remains unclear and respiratory infections, mostly viral, are typically proposed as potential triggers, although these are often not detected [184, 185]. Given emerging evidence of the role of ambient air pollution exposure (especially PM_2.5_) in increasing susceptibility to respiratory infections [186–188], a higher vulnerability to viral respiratory infections could be hypothesised as an intriguing mechanism through which air pollution can affect the risk of acute exacerbation in patients with IPF.

#### Gene–environment interaction

Given the known gene–environment interactions in IPF, some analyses have attempted to identify evidence of additive effects of air pollutants and genetic susceptibility on IPF risk. A polygenic risk score (PRS) for IPF was constructed using 13 susceptibility loci from a genome-wide association study [189] to evaluate the combined effect of air pollutants and genetic susceptibility on IPF risk. Participants with high genetic risk had a higher risk of incident IPF than those with low genetic risk. Therefore, Cui *et al*. [159] also evaluated the cumulative effect of genetic susceptibility on the relationship between long-term pollution exposures and IPF using the PRS. They analysed the associations of the *MUC5B* polymorphism alone and the PRS without the *MUC5B* polymorphism with the risk of incident IPF. Potential synergistic effects were found between NO_2_, NO_x_ and PM_2.5_ and the PRS on the risk of incident IPF, which were similar for both the *MUC5B* polymorphism and the non-*MUC5B* PRS [159]. Specifically, it has been hypothesised that a single-nucleotide polymorphism in the promoter of *MUC5B*, associated with increased risks of IPF, rheumatoid arthritis with ILD [190] and chronic hypersensitivity pneumonitis [191], may enhance susceptibility to alveolar damage by air pollution. MUC5B is involved in airway clearance and bacterial host defence, thus mucociliary dysfunction can cause higher retention of inhaled substances. To date, no influences have been demonstrated on the adverse effects of fine particles by *MUC5B* promoter status [192]. Interestingly, the *MUC5B* polymorphism has also been associated with better progression and survival in IPF [193, 194]. It could also be hypothesised that this polymorphism modulates the interactions between inhaled pollutants and cellular exposure.

## Future perspectives and research questions

Knowledge of the effects of air pollutants on alveolar health is lacking in many aspects, including the range of disease outcomes related to particulates and their precise causality. Determination of specific pollutant associations with disease end-points is complicated by the correlation between multiple pollutants and, indeed, it is notable that several of the studies above do not make use of multi-pollutant models in order to determine whether their exposure–outcome associations for specific pollutants are potentially markers of other relationships. Longitudinal, prospective studies are required to advance our understanding, including of gene–environment interactions. In parallel, mechanistic studies are required to better define how specific air pollutants perturb alveolar homeostasis. This knowledge has the potential to support targeted interventions in at-risk individuals.

To date, there are no established interventions to prevent ILA progression and only pharmacologic treatments have evidence of efficacy in progressive fibrotic lung diseases. Plausibly identifying and reducing air pollution sources and exposures offers the potential to modify the natural history of disease progression, so improving clinical outcomes. Changes in air quality policy, reducing airborne pollutant concentrations at source, represents the most effective and thus the best long-term goal, but also the most complex to implement.

Other preventive environmental interventions could include individual behavioural modifications for vulnerable patients with ILAs, such as reducing physical activity or staying indoors during times with higher ambient pollution concentrations. However, there is a growing appreciation of the importance of the potential health effects of indoor air quality, about which there has been much less research compared to outdoor air quality [195]. Air pollution indoors is a mixture of pollution from outdoors which has infiltrated into the indoor environment in a manner dependent on the location and permeability of the building and pollution generated within the indoor environment itself, from sources including cooking, combustion for heating, building and decorating materials, furnishings, cleaning products, personal hygiene products, pets, fungi, and humans themselves [196, 197]. Globally, household air pollution from solid fuels accounted for approximately 3.5 million deaths in 2010, but with a great deal of geographical heterogeneity [198]. However, the more recent Global Burden of Disease study has shown that there has been a marked decrease in global exposure to this risk [6]. Relatively few studies have examined the physical properties of indoor pollution and its sources compared to outdoors, and there is much less known about exposure at the personal level, such as could be provided by the use of wearable monitors capable of capturing aspects of the indoor environment [199, 200]. There are moves towards a better understanding of the sources and composition in-home air pollution. In the UK, there has been a recent move towards larger, consortium-based projects, such as the UnderstandING the sourcEs, traNsformations and fates of IndOor air pollUtantS (INGENIOUS) [201] and West London Healthy Home and Environment (WellHome) studies [202]. These were both funded under the large United Kingdom Research and Innovation (UKRI) Clean Air Strategic Priorities Fund [203], as were the Hazard Identification Platform to Assess the Health Impacts from Indoor and Outdoor Air Pollutant Exposures, through Mechanistic Toxicology (HIPTox) programme [204] and Relating Environment-use Scenarios in Pregnancy/Infanthood and Resulting airborne material Exposures to child health outcomes (RESPIRE) study. A better understanding of indoor air pollution is crucial, given that people spend the great majority of their time indoors and, as such, the indoor environment can make a substantial contribution to the exposome, similar to contributions from occupational and transport microenvironments [41, 205, 206]. Separately, two recently UKRI-funded transdisciplinary research hubs will focus on indoor air quality and how it might be affected by moves towards net zero – the Child And Adolescent Health Impacts Of Learning Indoor Environments Under Net Zero (Chili Hub) [207] and Indoor HABItability during the Transition to Net Zero Housing Hub (INHABIT) [208]. Moves towards these larger, multi-disciplinary, multi-institutional consortia perhaps suggest the complexity of the issues surrounding air quality, especially indoor air quality, and of the approaches needed for its study and mitigation.

Other preventive options may include replacing modes of transportation, cleaning indoor air with high-efficiency particulate air filters and using respirators or other types of personal face masks to reduce PM exposure (*e.g.* N95 respirators in the US and FFP2 respirators in Europe) [209, 210]. However, there is a paucity of studies demonstrating the efficacy of such measures in patient populations. Furthermore, there is a need to explore the effects of environmental and behavioural modification recommendations in mitigating the risk of exposure to indoor and outdoor air pollution and, consequently, the risk of interstitial lung abnormalities progression in clinical lung fibrosis.

Additionally, further research is needed to better assess a potentially different impact of air pollution on the natural history of non-IPF fibrotic ILDs, given previous epidemiological studies are largely focused on IPF, and data on the risk factors for development and acute exacerbations of other forms of pulmonary fibrosis are largely unexplored.

Finally, there is a growing awareness of the need to understand what climate change may mean for air quality and its effects on health [211]. Aridity and atmospheric instability have been associated with increased risk of wildfires [212, 213], while increased temperatures and altered land use have also resulted in drying of lake beds, facilitating the generation and suspension of geogenic PM [214–216]. Increased temperatures are also associated with increased formation of biogenic volatile organic compounds (BVOCs) [217]. BVOCs play an important role in the formation of tropospheric O_3_ [218]; consequently, global O_3_-associated mortality has been predicted to rise by 48–70% from 2000 to 2090 [219]. There is also some evidence that increased temperatures may increase the effects on health of air pollution exposure, at least for asthma and COPD [220]. However, there is no evidence that the authors are aware of in the current literature evidencing direct interactive effects of climate change and pollution on lung fibrosis.

## Conclusions

There is increasing evidence that air pollution exposure perturbs alveolar homeostasis, promoting the development and progression of clinically significant lung fibrosis (summarised in table 1). Prospective studies are required to better define the role of specific pollutants, including those in the indoor environment, in the development and progression of ILDs to clinically significant fibrotic ILDs, as well as the role of gene–environment interactions in these effects. Ultimately, the ability to better identify at-risk individuals may offer the possibility to support targeted interventions in individuals with ILAs, thereby reducing the likelihood of development of clinically significant lung fibrosis.

**TABLE 1:** Summary of cited studies reporting associations between major air pollutants and alveolar changes

Air pollutants	Potential alveolar modifications							
	Alveolar epithelial injuries	Telomere shortening	Epigenetic changes	Changes in microRNA expression	ILAs/HAAs	Fibrosis development	Fibrosis progression	AE-IPF
**PM_10_**		[116]; no association: [118]^#^	[134]^¶^, [136]	[142]		[109]^+^, [159]^§^, [164]	[163], [181]	[183]
**PM_2.5_**	[94], [104]^ƒ^	[84], [116], [117] (black carbon); no association: [118]^#^, [119]	[128]^##^, [131], [134]^¶^, [135]	[142]^¶¶^, [144], [148]	[154], [157]^++^	[108], [159], [160], [161], [162]^§§^, [164]	[170], [177]^ƒƒ^, [181], [192]	[169], [182], [183]
**PM_0.1_**	[96]^###^			[142]			[178]	
**NO**								[182]
**NO_2_**		No association: [118]	[135]	[142]	[154]	[158], [159], [160], [164]	[166]	[169], [180], [183]
**NO_x_**		No association: [118]			[154]	[159]	[166]	
**O_3_**	[106], [107]		[134]^¶^			[106], [107], [167]	[166], [167]	[180], [181]
**CO**			[135]				[166]	

Empty cells indicate either lack of evidence or areas where further studies are needed. “No association” refers to papers where no association with any investigated air pollution metric is found. For papers where some, but not all, investigated pollutants are significantly associated with an endpoint, only these associated pollutants are shown in this table. Reference numbers correspond to studies cited in the manuscript's reference list. AE-IPF: acute exacerbation of idiopathic pulmonary fibrosis; CO: carbon monoxide; HAA: high attenuation area; ILA: interstitial lung abnormality; NO: nitric oxide; NO_2_: nitrogen dioxide; NO_x_: nitrogen oxides; O_3_: ozone; PM: particulate matter; PM_10_: coarse particulate matter; PM_2.5_: fine particulate matter; PM_0.1_: ultrafine particulate matter. ^#^: Also found no association for PM_2.5_ absorbance and PM_coarse_ (*i.e.* PM_10–2.5_). ^¶^: Evaluated differences between more and less polluted cities, evidencing this comparison with PM_10_, PM_2.5_ and O_3_ measurements, but did not formally evaluate associations with the pollutants. ^+^: Uses PM dissolved in organic solvent. PM is NIST SRM1649b, which has a mean aerodynamic diameter of 24.3 µm. Therefore, this is larger than PM_10_, but is included here with this caveat, given that a portion of the sample lies within the PM_10_ fraction. ^§^: Association between PM_10_ and incident idiopathic pulmonary fibrosis was significant only when comparing participants with high and low polygenic risk scores. ^ƒ^: Performed in human embryonic fibroblasts, but is included for completeness. ^##^: PM_2.5_ (model dependent) along with PM_2.5_ sulfate, nitrate, ammonium and black carbon. ^¶¶^: PM_2.5_ and, separately, black carbon. ^++^: Association between elemental carbon and risk of ILA and ILA progression, but no association for PM_2.5_ *per se*. ^§§^: PM_2.5_ components contributing most to risk were ammonium, mineral dust and black carbon. ^ƒƒ^: PM_2.5_ and PM_2.5_ sulfate, ammonium, and black carbon. ^###^: Does not demonstrate epithelial injury, but rather suggests that injured epithelium may be more susceptible to translocation of carbonaceous ultrafine PM.
